# A Multicenter, Double-Blind, Randomized, Placebo-Controlled Trial to Evaluate the Efficacy and Safety of Duliang Soft Capsule in Patients with Chronic Daily Headache

**DOI:** 10.1155/2015/694061

**Published:** 2015-05-26

**Authors:** Shengyuan Yu, Yueqing Hu, Qi Wan, Jiying Zhou, Xinfeng Liu, Xiangyang Qiao, Xiaosu Yang, Jiachun Feng, Kangning Chen, Xiaoping Pan, Qingwu Yang, Linsen Dou, Ming Liu, Yangmei Chen, Tingmin Yu, Juming Yu, Zhiwei Li, Xue Bai, Jingfeng Duan

**Affiliations:** ^1^Department of Neurology, Chinese PLA General Hospital, Beijing 100000, China; ^2^Department of Neurology, The First Affiliated Hospital, Nanjing Medicine University, Nanjing, Jiangsu 210000, China; ^3^Department of Neurology, The First Affiliated Hospital of Chongqing Medical University, Chongqing 400000, China; ^4^Department of Neurology, Nanjing General Hospital of Nanjing Military Command, Nanjing, Jiangsu 210000, China; ^5^Department of Neurology, Affiliated Huashan Hospital of Fudan University, Shanghai 200000, China; ^6^Department of Neurology, Xiangya Hospital of Central South University, Changsha, Hunan 410000, China; ^7^Department of Neurology, The First Hospital of Jilin University, Changchun, Jilin 130000, China; ^8^Department of Neurology, Southwest Hospital, Third Military Medical University, Chongqing 400000, China; ^9^Department of Neurology, First People's Hospital of Guangzhou, Guangzhou, Guangdong 510000, China; ^10^Department of Neurology, Xinqiao Hospital, Third Military Medical University, Chongqing 400000, China; ^11^Department of Neurology, Beijing Tiantan Hospital, Capital Medical University, Beijing 100000, China; ^12^Department of Neurology, West China Hospital, Sichuan University, Chengdu, Sichuan 610000, China; ^13^Department of Neurology, The Second Affiliated Hospital of Chongqing Medical University, Chongqing 400000, China; ^14^Department of Neurology, The Second Hospital of Jilin University, Changchun, Jilin 130000, China; ^15^Department of Neurology, Affiliated Hospital of North Sichuan Medical College, Nanchong, Sichuan 637000, China; ^16^Department of Neurology, Yongchuan Hospital of Chongqing Medical University, Chongqing 400000, China; ^17^Department of Neurology, TCM Hospital of Luzhou Medical College, Luzhou, Sichuan 646000, China; ^18^Department of Neurology, Mianyang Central Hospital, Mianyang, Sichuan 621000, China

## Abstract

*Objective*. To investigate the efficacy and safety of traditional Chinese medicine Duliang soft capsule (DSC) in prophylactic treatment for patients with chronic daily headache (CDH). *Methods*. A multicenter, double-blind, randomized, placebo-controlled clinical study was conducted at 18 Chinese clinical centers. The participants received either DSC or placebo for 4 weeks. The primary efficacy measure was headache-free rate (HFR) in a 4-week period between the pretreatment and posttreatment stages. The secondary efficacy measures were the decrease of headache days, the duration of headache attacks, the frequency of analgesic usage, quality of life, disability, and the headache severity (VAS scores). The accompanying symptoms and adverse events were also assessed. *Results*. Of 584 CDH patients assessed, 468 eligible patients were randomized. 338 patients received DSC, while 111 patients were assigned in the placebo group. Following treatment, there was a 16.56% difference in HFR favoring DSC over placebo (*P* < 0.01). Significant differences were also observed between DSC and placebo groups in the secondary measures. However, no statistical difference was found between the two groups in the associated symptoms. No severe adverse effects were observed in the study.* Conclusions*. DSC might be an effective and well-tolerated option for the prophylactic treatment of patients with CDH.

## 1. Introduction

Chronic daily headache (CDH) is defined by the presence of a headache 15 days or more per month for longer than 3 months according to the third edition of the International Classification of Headache Disorders (ICHD-3 beta) [1]. CDH is not a diagnosis but a category that contains many disorders representing primary and secondary headaches. Primary CDH can be subdivided into disorders of long duration (>4 h/attack) and disorders of short duration (<4 h/attack). CDH disorders of long duration include chronic migraine (CM), chronic tension-type headache (CTTH), hemicrania continua (HC), and new daily persistent headache (NDPH). CDH usually consists of a mixture of migraine and tension-type headaches (TTH). Some patients have pure chronic TTH and no migrainous features, and others have only migraine, but most have a mixed migraine-TTH pattern [2]. Approximately 1% to 5% of the population worldwide suffers from CDH but accounts for more than 40% of presentations to headache-specialty clinics [3–5].

The disability associated with CDH is substantial and includes a diminished quality of life (QOL) related to physical and mental health, as well as impaired social and occupational function [6–8]. Over half of all patients with CDH have sleep disturbances and mood disorders such as depression or anxiety [9], and these disorders can exacerbate the underlying headache. The prophylactic treatment of CDH might play an important role in breaking the vicious circle. However, there have been few well-conducted trials examining the prophylactic treatment of CDH [10]. The previous studies indicated that some agents might have some benefits in CDH but failed to reach the main efficacy of prophylactic treatment, such as gabapentin [11], valproate [12, 13], levetiracetam [14], and antidepressants like tizanidine [15], paroxetine [16], olanzapine [17], fluoxetine [18], and amitriptyline [19]. And to date, only botulinum toxin A [20, 21] and topiramate [22] have large properly conducted placebo-controlled trials with positive outcome in subjects with chronic migraine. Given that CDH is a highly disabling disorder with frequently unsatisfactory treatment outcomes, there appears to be an urgent need to explore new medications.

Traditional Chinese medicine (TCM) has played an important role in the medical care of headache for thousands of years in China. For example, Du Liang Prescription has a long history of the treatment of headache in the traditional Chinese pharmacopoeia* Bai Yi Xuan Fang* (selected formulas) by Wang Miu since the Song Dynasty about 1196 A.D. It contains* Radix Angelica dahurica* and* Ligusticum chuanxiong* by a ratio of 4 : 1.* Angelica dahurica* has been widely used in TCM for the treatments of headache, toothache, acne, ulcer, and carbuncle [23], while* Ligusticum chuanxiong* has been used for treatments of migraine and cardiovascular diseases in China for centuries [24]. Duliang soft capsule (DSC) is a Chinese herbal medicine (CHM) made according to Du Liang Prescription and has been approved by the China State Food and Drug Administration (Authorized Document number Z20000011) for the treatment of headache since 2000. To date, researchers have revealed several active ingredients in DSC. Furanocoumarin may be the active modulation for the transient receptor potential vanilloid type 1 (TRPV1) channel, an important transmission of nociceptive information [25].* Radix Angelica dahurica* extracts that reinforce the analgesic effects were found to be related to the improvement of the plasma concentration of dL-THP [23]. Senkyunolide I (SEI), a primary metabolite of* Ligusticum chuanxiong*, was shown to display antimigraine effect, and the mechanisms of pain relief in migraine model rats may be through adjusting the levels of monoamine neurotransmitters and their turnover rates, as well as decreasing nitric oxide levels in the blood and brain [26–28]. Based on the above evidences, DSC combined the analgesic effects of* Radix Angelica dahurica* with the modulation of* Ligusticum chuanxiong* for blood and brain to relieve the headache. There were some small trials of DSC suggesting benefit in migraine and tension-type headache [29–31]. It follows that DSC may prove efficacious in the prophylaxis of CDH. We therefore designed and conducted this multicenter, double-blind, randomized, placebo-controlled clinical trial to evaluate the safety and efficacy of DSC for the prophylactic treatment of CDH.

## 2. Subjects and Methods

### 2.1. Standard Protocol Approvals

The study protocol was complied with the World Medical Association Declaration of Helsinki and China's regulations and guidelines on good clinical practice. Ethical clearance for the trial was obtained from the Ethical Committee of the Chinese PLA General Hospital. All participating centers obtained approval of their local Ethical Review Board. Informed consents were obtained from all participants before the study.

### 2.2. Subjects

Patients who were complaining of chronic headache were randomly recruited, and then they underwent the screening course to select the subjects who met the following inclusion criteria: (a) subjects aged between 18 and 65 years old; (b) those who have a diagnosis of CM and CTTH according to ICHD-2 diagnostic criteria; (c) all subjects willing and able to sign an informed consent before the study; (d) all patients experiencing headaches on 15 days or more per month for longer than 3 months; (e) patients willing to complete the entire course of the study and comply with study instructions.

Exclusion criteria included (a) secondary headaches except for probable medication overuse with preexisting migraine or tension-type headache; (b) serious comorbidities including cardiovascular, hepatic, renal, cerebellar, endocrine, and hematologic diseases and malignant tumors, psychosis, epilepsy, or glaucoma; (c) use of antidepressants, antiepileptics, calcium-channel blockers, *β*-adrenergic receptor blockers, or other traditional Chinese medicines indicated for headache and taken up to two weeks prior to the trial; (d) participation in other clinical trials; (e) having a history of drug or food allergy or being allergic to a component of DSC; (f) pregnant and lactating women or those planning to become pregnant; and (g) participants who never took in the DSC capsules or placebo and never recorded their headaches.

### 2.3. Study Design

**Figure 1:**
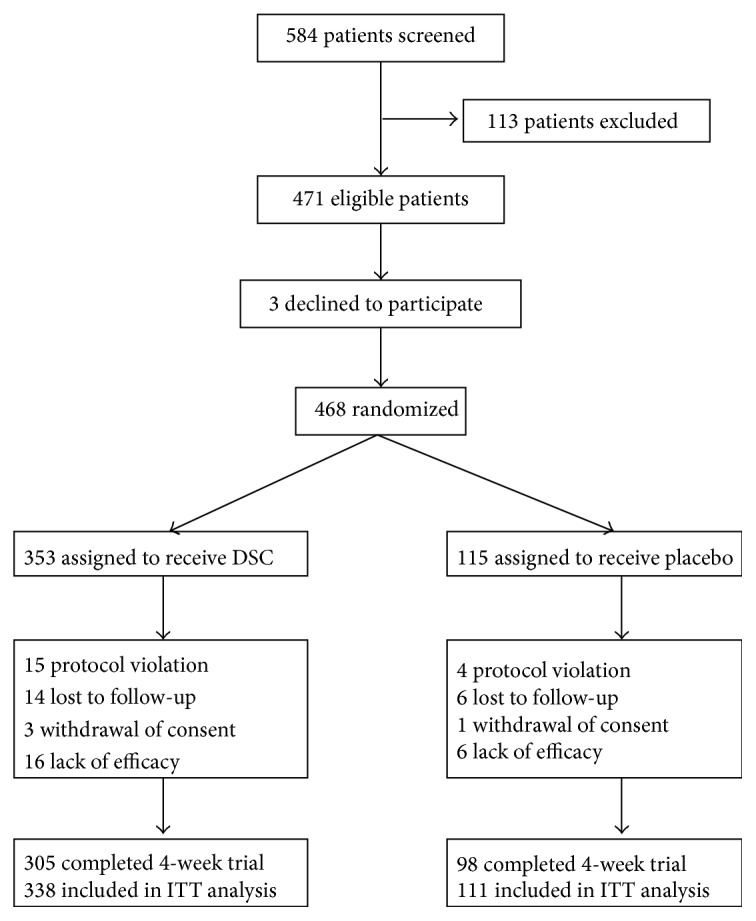
Flow diagram illustrating the number of patients in each group throughout the study.

This was a 4-week, multicenter, randomized, double-blind, placebo-controlled, pilot study of DSC in the prophylaxis of CDH at 18 centers in China. The study was approved by each center's human research ethics committee. The Chinese PLA General Hospital was the responsible department for design and execution of the clinical trial. This trial was registered with Chinese Clinical Trial Register (ChiCTR-TRC-13003459). A total of 584 patients were screened, among which 113 patients were excluded. In total, 471 eligible CDH patients were recruited and 468 were randomized in a 3 : 1 ratio to receive DSC or placebo treatment. The detailed flow diagram for screening course was shown in Figure 1 and the efficacy and safety of DSC were assessed after the 4-week treatment. Randomization was external to participating centers. The randomization was performed using permuted blocks according to standardized operating procedures. Computer-generated random medication code numbers were prepared and preprinted on the study medication labels. The investigators entered the eligible patient's identifier in numerical order.

The present study consisted of a baseline period lasting 4 weeks and a treatment period of 4 weeks. Eligible patients were evaluated based on self-reported history, physical examinations, and laboratory tests. From the baseline period, eligible patients were instructed not to take preventive medications and to maintain a headache diary. Following the baseline period, patients meeting the inclusion criteria were randomized 3 : 1 to DSC or placebo. The dosage used in this study was 3 capsules of DSC or placebo 3 times daily (486 mg/d). Drugs were given with the same identical color and shape. Patients needed to record their headache symptoms and the usage of acute headache medications, which were collected by a standard questionnaire once every week at the 18 headache clinics and by their headache diary. The efficacy and safety of DSC were assessed after the 4-week treatment.

Because many patients with CDH are refractory to all kinds of intervention and their lack of response could conceal efficacy in others, the commonly accepted criteria of efficacy for headache prophylaxis, a 50% reduction in frequency of headache, were considered inappropriate for this highly refractory headache form [11]. Based on two previous trials of gabapentin and LEV in CDH, a difference of 7.5% in the primary efficacy parameter, headache-free rate (HFR), was adopted for this study [10, 14]. A mean difference of 7.5% would be detected at the 5% level with 80% power if at least 70 subjects were enrolled. For the sake of attrition, a target sample size of at least 120 patients was selected. In order to assess the safety of DSC in CDH patients better, the DSC group size was enlarged to 360 patients.

### 2.4. Efficacy Measures

The primary efficacy measure was HFR for the 4-week treatment period. HFR was calculated by the formula HFR = (*R*/*N*)%, where *R* represented the number of headache-free days during treatment period. *N* was 28 days. A headache-free day was defined as a complete day (clock time of 00:00 to 24:00) in which no headache was recorded.

The secondary efficacy parameters included decrease of headache days, headache duration, associated symptoms, severity of pain using a visual analogue scale (VAS), frequency of analgesic usage, and results from Short Form-36 QOL questionnaires and disability (Headache-Attributed Lost Time) [32].

### 2.5. Safety Measures

Safety was assessed by reports of adverse events, physical and neurological examinations, and clinical laboratory tests (complete blood count, urinalysis, and serum chemistry profile). The adverse events were recorded and documented with information regarding the date of onset, resolution date, severity, duration, frequency, relationship to study treatment, action taken, and outcome.

### 2.6. Statistical Analysis

Analyses of efficacy were performed on the intent-to-treat population (full analysis set). Safety analyses were performed on all randomized subjects who received at least 1 dose of study medication and at least 1 posttreatment safety measurement. All statistical analyses were performed with SAS software, version 9.1.3 (SAS Institute, Cary, NC, USA). For the nonnormality of the data, a nonparametric signed rank test was performed. Continuous variables were presented as the mean ± SD and paired* t*-test was performed. The chi-square test or Wilcoxon test was adopted for categorical variables when appropriate.* P* values <0.05 were considered statistically significant, and all tests were 2-tailed.

## 3. Results

From February 2011 to September 2013, in total, 471 Chinese CDH patients were recruited and 468 were randomized in a 3 : 1 ratio to receive DSC or placebo treatment for 4 weeks (DSC: *n* = 353; placebo: *n* = 115). The detailed flow diagram for screening course was shown in Figure 1. All 468 randomized patients were eligible for the safety analysis. The intent-to-treat population consisted of 449 subjects (DSC: *n* = 338; placebo: *n* = 111).

Demographic and baseline headache characteristics of both groups were shown in Table 1. No significant differences were observed between the two groups at baseline. There was no significant difference of the HFR at baseline between DSC group (19.11 ± 19.25%) and placebo group (16.7 ± 18.6%) (*P* = 0.31), indicating a refractory headache profile.

**Figure 2:**
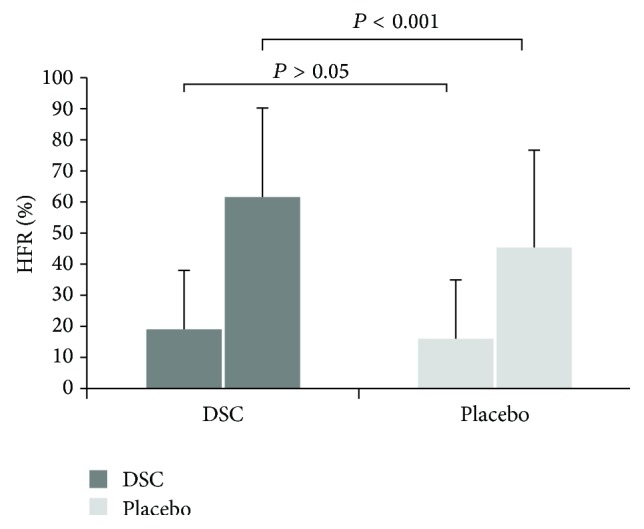
The change of HFR between DSC and placebo groups in the pretreatment and posttreatment stages.

The primary endpoint was the difference in the percentage of days during which headaches occurred while on DSC compared with placebo, which was calculated by the percentage of headache-free days. After 4-week treatment, Figure 2 showed that HFR was 61.93 ± 27.62% in the DSC group and 45.37 ± 30.6% for the placebo group. The difference was 16.56%, favoring DSC group (*P* < 0.01).

Compared with the placebo group, the headache duration, analgesic usage, VAS scores of pain, and disability decreased in DSC group (*P* < 0.01), as shown in Table 2. DSC treatment significantly improved QOL as measured by changes in the eight SF-36 domains: (i) physical function (*P* < 0.01); (ii) role-physical (*P* < 0.01); (iii) social function (*P* < 0.01); (iv) bodily pain (*P* < 0.01); (v) vitality (*P* < 0.01); (vi) mental health (*P* < 0.01); (vii) role-emotional (*P* < 0.01); and (viii) general health (*P* < 0.01).

There was significant difference in the accompanying symptoms of headache between the pretreatment and posttreatment stages for both DSC and placebo groups, including nausea, vomiting, photophobia, phonophobia, and dizziness, which decreased obviously after 4 weeks of treatment (*P* < 0.05, shown in Table 3). However, no significant difference was found in these associated symptoms between DSC group and placebo group after treatment (Table 4, *P* > 0.05). During the trial, no clinically relevant changes in mean laboratory test values were observed for either treatment group. No deaths or serious adverse events were reported during the study. A total of 13 adverse events were reported. Mild-to-moderate adverse events were reported in both treatment group and placebo group (Table 5). Eight AEs occurred on DSC, while 5 AEs occurred on placebo. Adverse events that possibly related to treatment were reported in 2 patients (0.59%) in DSC group complaining of mild nausea, which occurred in 1 patient (0.90%) in placebo group. Mild gastric discomfort was reported in 1 patient in DSC group and moderate weakness was recorded in 1 patient in placebo group. All the symptoms disappeared in a few days and did not disturb the continuation of the trial.

## 4. Discussion

This study firstly showed that the traditional Chinese medicine DSC could effectively treat CDH by a multicenter randomized double-blind controlled trial. Prophylactic pharmacological treatment of CDH has been studied in previous studies, but only the botulinum toxin A and topiramate have satisfactory efficacy [10]. Several reasons may contribute to the failure of other medications, including CDH's high resistance to medications and lack of diagnostic criteria of CDH. Furthermore, the systemic side effects of these medications also limit their usefulness as prophylactic treatments. Keller et al. reported that 11 of 93 patients treated with paroxetine had serious adverse events compared with 2/87 in the placebo group [33]. Among 373 patients in the trial by Wagner et al., 9% (17/189) treated with sertraline withdrew because of adverse events, compared with 3% (5/184) in the placebo group. Wagner et al. concluded 7 adverse effects that occurred in at least 5% of the sertraline group, at least twice as often as in the placebo [34]. Even though topiramate was proved to be effective to treat CDH, its discontinuation rate was around 25% in clinical trials due to AEs. Paraesthesia was the most common AE although cognitive impairment was the main reason for discontinuation [35]. In fact, adverse effects might be more frequent in clinic than the authors of these studies implied. Therefore other drug options should be considered.

As* WHO Traditional Medicine (TM) Strategy 2014–2023* suggests that traditional medicine stands out as a way of coping with the relentless rise of chronic noncommunicable diseases, nearly a quarter of all modern medicines are derived from natural products, many of which were first used in a traditional medicine context. TM is thus a resource not only for primary health care, but also for innovation and discovery. DSC is such a traditional medicine. It contains a compound preparation of* Radix Angelica dahurica* and* Ligusticum chuanxiong*, which have been commonly used in Chinese people's daily life from ancient China.* Radix Angelica dahurica* is widely used to treat headache, toothache, and orbital eye pain, and* Ligusticum chuanxiong* is a popular Chinese medicinal herb used for treatment of migraine and cardiovascular diseases in China for centuries [36]. It can be viewed as some kind of large-scale real life trial demonstrating good safety of these two herbs. It could partly explain why the AEs reported in DSC group were limited compared with the abovementioned trials. Meanwhile, no somnolence was reported during treatment, which is very common in almost all prophylactic medications of headache. Potentially, it implies DSC has significant value for professional drivers and machine operators suffering from CDH. Another reason for the low percentage of AE in this trial may be that the treatment duration was designed as one month rather than 3 to 6 months adopted in other trials. When discussing the protocol of this pilot study, researchers were canvassed and determined the one-month duration of medication, considering the culture and compliance of Chinese headache patients. In the future long-term clinical trials are needed to further study DSC in treating CDH.

The current study demonstrated that DSC significantly increased the percentage of HFR and improved the related symptoms. Compared with placebo, the mean of 16.56% increase in HFR on DSC (*P* < 0.01) exceeded the selected criterion to demonstrate clinical relevance, thus confirming its benefit in the prophylaxis of CDH. Furthermore, the headache days reduced from 22.65 ± 5.39 days per month to 10.66 ± 7.73 days per month in DSC group, while the headache days changed from 23.32 ± 5.21 days to 15.3 ± 8.57 days per month in placebo group. The DSC group achieved a 52.9% reduction in frequency of headache after treatment, favoring DSC (*P* < 0.01). There were no severe adverse events during the study. No hepatic and renal function damage was observed. The most common adverse events were temporary nausea and gastric discomfort.

Further benefits were observed in the present study. DSC was superior to placebo in terms of reduction in headache frequency, severity, and disability, and an enhanced QOL. As a consequence to these benefits, there was a reduction in analgesic usage.

Similar to many trials in CDH [37, 38], a significant placebo effect was demonstrated in this study. After treatment, the nausea, vomiting, photophobia, phonophobia, and dizziness were improved significantly from baseline on DSC (*P* < 0.01) but still failed to achieve statistical significance compared with placebo. In primary headache trials, the placebo effect is quite complex, probably because of its close relation with psychological problems. This emphasizes the need for a placebo arm in all headache trials.

Several potential limitations of the present study should be addressed. Firstly, the follow-up course is relatively short (only 4 weeks). Secondly, the study protocol excluded the high risk population and those patients with severe complications; it only reflected the features of certain important population of CDH in Chinese population. Thirdly, since many diagnosis and evaluation methods of headache are subjective (i.e., VAS, QOL, etc.), ideally a core lab should be set so that the symptoms can be judged by the same group of doctors, but as participating patients in our study came from 18 test centers across the country; it is impractical to transport them to one hospital. Fourthly, the results of the present study demonstrated a significant placebo response in CDH, which reminded us of the treatment of CDH that hence should be evaluated using stringent controlled-trial methodology.

In summary, this trial demonstrated that DSC 486 mg/day significantly reduced monthly headache days compared with placebo and was a safe and well-tolerated preventive therapy in this group of subjects with CM or CTTH. DSC can alleviate the CDH-related symptoms and improves the quality of life of CDH patients. The results of the study suggested that traditional Chinese medicine DSC was safe and effective for treating patients with CDH. More comprehensive studies need be carried out in the future.

## Figures and Tables

**Table 1:** Baseline characteristics of patients receiving Duliang soft capsules (DSC) and placebo (intent-to-treat population).

Characteristics	DSC (*n* = 338)	Placebo (*n* = 111)	*P* values
Age (years)	43.64 ± 11.38	44.56 ± 11.36	0.46
Height (cm)	162.12 ± 7.17	163.93 ± 7.11	0.05
Weight (kg)	60.61 ± 11.06	62.05 ± 15	0.28
Body temperature (°C)	36.57 ± 0.27	36.61 ± 0.27	0.18
Pulsation (bpm)	72.78 ± 7.47	74.17 ± 8.43	0.10
Systolic blood pressure (mmHg)	120.75 ± 10.32	120.46 ± 11.8	0.81
Diastolic blood pressure (mmHg)	76.43 ± 7.62	74.98 ± 9.05	0.10
Breathing (bpm)	18.44 ± 1.5	18.39 ± 1.4	0.74
BMI (kg/m^2^)	23.03 ± 3.74	23 ± 5.07	0.95
VAS score, *n* (%)			
Mild (0–3)	75 (22.19%)	33 (29.73%)	0.69
Moderate (4–6)	146 (43.2%)	37 (33.33%)	0.08
Severe (7–10)	117 (34.62%)	41 (36.94%)	0.76
Site of headache, *n* (%)			
Unilateral	102 (30.18%)	40 (36.04%)	0.06
Bilateral	195 (57.69%)	59 (53.15%)	0.19
Others	41 (12.13%)	12 (10.81%)	0.45
Use of analgesic, *n* (%)			0.34
Yes	333 (98.52%)	111 (100%)	
No	5 (1.48%)	0 (0)	
Headache duration per day, *n* (%)			0.88
≥4 h	235 (69.53%)	78 (70.27%)	
<4 h	103 (30.47%)	33 (29.73%)	
Headache type at screening			
Migraine	72 (21.30%)	23 (20.72%)	0.79
Tension-type headache	84 (24.85%)	29 (26.13%)	0.60
Both migraine and TTH	149 (44.08%)	48 (43.24%)	0.75
Others	33 (9.76%)	11 (9.91%)	0.93

**Table 2:** The secondary efficacy measures between pretreatment and posttreatment stages.

Variables	Pretreatment		*P*1 value	Posttreatment		*P*2 value
	DSC (*n* = 338)	Placebo (*n* = 111)		DSC (*n* = 338)	Placebo (*n* = 111)	
Headache days (m)	22.65 ± 5.39	23.32 ± 5.21	0.30	10.66 ± 7.73	15.3 ± 8.57	<0.01
Headache duration (d)	9.4 ± 8	9.8 ± 8.14	0.73	4.62 ± 5.92	6.25 ± 6.7	<0.01
Analgesic usage	5.58 ± 2.99	5.09 ± 2.71	0.15	2.47 ± 2.17	3.36 ± 2.88	<0.01
Severity of pain (VAS)	5.54 ± 2.1	5.56 ± 2.41	0.91	2.5 ± 1.89	3.42 ± 2.37	<0.01
Disability	2.18 ± 1.41	2.36 ± 1.7	0.55	0.69 ± 1.06	1.27 ± 1.55	<0.01

**Table 3:** Change in accompanying symptoms from baseline to after 4 weeks of treatment in both groups.

Symptoms	DSC group *n* (%)		*P*1 values	Placebo group *n* (%)		*P*2 values
	Pretreatment	Posttreatment		Pretreatment	Posttreatment	
Nausea	135 (39.94%)	57 (16.68%)	<0.01	43 (38.74%)	14 (12.61%)	<0.01
Vomiting	60 (17.75%)	27 (7.99%)	<0.01	29 (26.13%)	8 (7.21%)	<0.01
Photophobia	124 (36.69%)	60 (17.15%)	<0.01	35 (31.53%)	23 (20.72%)	0.01
Phonophobia	207 (61.24%)	119 (35.21%)	<0.01	60 (54.05%)	35 (31.53%)	<0.01
Dizziness	197 (58.28%)	126 (37.28%)	<0.01	73 (65.77%)	49 (44.14%)	<0.01

**Table 4:** Change in accompanying symptoms between DSC and placebo groups after treatment.

Symptoms	DSC group *n* (%)	Placebo group *n* (%)	*P* values
Nausea	57 (16.86%)	14 (12.61%)	0.29
Vomiting	27 (7.99%)	8 (7.21%)	0.79
Photophobia	60 (17.75%)	23 (20.72%)	0.48
Phonophobia	119 (35.21%)	35 (31.53%)	0.48
Dizziness	126 (37.28%)	49 (44.14%)	0.20

**Table 5:** Adverse events in this study.

Item	DSC			Placebo			Relatedness to drugs
	Mild	Moderate	Severe	Mild	Moderate	Severe	
Common cold	1	1		1	1		N
Constipation					1		N
Pelvic inflammation or vaginitis	1						N
Pharyngodynia		1					N
Vaginal bleeding		1					N
Nausea	2			1			P
Hypodynamia					1		P
Gastric discomfort	1						P

*Note*. N: not related. P: possibly related.
